# Effects of foliar application of salicylic acid and nitric oxide in alleviating iron deficiency induced chlorosis of *Arachis hypogaea L.*

**DOI:** 10.1186/1999-3110-55-9

**Published:** 2014-01-20

**Authors:** Jing Kong, Yuanjie Dong, Linlin Xu, Shuang Liu, Xiaoying Bai

**Affiliations:** College of Resources and Environment, Shandong Agricultural University, Tai’an, 271018 China

**Keywords:** Active iron, Antioxidant enzymes, *Arachis hypogaea* L., Mineral elements, SA, SNP

## Abstract

**Background:**

The aim of this experiment was to analyze the alleviation mechanism of exogenous salicylic acid (SA) and sodium nitroprusside (SNP, a nitric oxide donor) on peanut seedlings under Fe deficiency. The effects of SA and SNP on iron uptake and availability, ions balance and oxidant damage were studied with foliar application of exogenous 1.0 mM SA (SA) or 2.5 mM SNP (SNP) or 0.5 mM SA+1.25 mM SNP [1/2(SA+SNP)] or 1.0 mM SA+2.5 mM SNP (SA+SNP).

**Results:**

The results showed that after 21 days treatment, the peanut seedlings growing under iron deficiency conditions exhibited leaf interveinal chlorosis, and this iron-deficiency induced symptom was prevented by foliar application of SA, SNP, 1/2 (SA+SNP), especially SA+SNP. The increased contents of chlorophyll and active iron, and increased Fe accumulation in cell organelles were observed in SA+SNP treated young leaves, suggesting that an improvement of iron availability in plants. Moreover, the improved nutrient solution pH, increased H^+^-ATPase activity and increased iron concentration in roots in SA+SNP treated plants, suggesting that SA+SNP is effective in modulating iron uptake. Furthermore, the increased calcium (Ca), magnesium (Mg) and zinc (Zn) concentrations and decreased manganese (Mn) and copper (Cu) concentrations in the leaves and roots of peanut indicated that SA+SNP stimulated the maintenance of ions disturbed by Fe deficiency. In addition, SA+SNP alleviated the increased accumulation of superoxide anion (O_2_^•-^) generation rate and malondialdehyde (MDA), and modulated the antioxidant enzymes.

**Conclusions:**

These results indicated that the interaction of SA and SNP promoted Fe uptake, translocation and activation; modulated the balance of mineral elements; and protected Fe deficiency induced oxidative stress. Therefore, SA and SNP had synergistic effects in alleviating chlorosis induced by Fe deficiency.

**Electronic supplementary material:**

The online version of this article (doi:10.1186/1999-3110-55-9) contains supplementary material, which is available to authorized users.

## Background

Iron (Fe) is an essential mineral nutrient for plants. It acts as a co-factor for many enzymes and proteins. Fe is involved in chlorophyll biosynthesis, thylakoid synthesis, and chloroplast development (Buchanan et al. 2000). Therefore, Fe deficiency impairs chlorophyll biosynthesis and chloroplast development in both dicotyledonous and monocotyledonous species (Graziano et al. 2002). Additionally, in higher plants, it is well known that the retranslocation of Fe from old leaves to young leaves is difficult. Therefore, when the plants suffer from Fe deficiency, the newly forming leaves develop chlorosis symptoms (Jin et al. 2007). Peanuts belong to Strategy I plants, and it responds to Fe deficiency by absorbing iron in three successive steps. First, they acidify the rhizosphere to increase the solubility of Fe(III) (Santi and Schmidt 2009). Next, they reduced soluble Fe(III) to Fe(II) by the ferric-chelate reductase FRO2 (Ding et al. 2009). Finally, plants transport Fe(II) across the root plasma membrane by the metal transporter IRT1 (Ding et al. 2010). However, the factors that induced Fe-deficiency chlorosis of peanut were very complicated in the field, and the best predictor of Fe efficiency in peanut was not very clear (Gao and Shi 2007).

Salicylic acid (SA) is an endogenous growth regulator of phenolic nature, which participates in the regulation of physiological processes and plant resistance to biotic and abiotic stress (Karlidag et al. 2009). For example, it improves germination, plant growth, transpiration rate, stomatal regulation and photosynthesis, ion uptake and transport (Khan et al. 2003; Metwally et al. 2003; Khodary 2004; He et al. 2010). Furthermore, it is now clear that SA provides protection against a number of abiotic stress as heat stress in strawberry (Karlidag et al. 2009), heavy metal stress such as cadmium (Metwally et al. 2003; He et al. 2010), copper (Khodary 2004; El-Tayeb 2006) and manganese (Shi and Zhu 2008), and salt stress (El-Tayeb 2005; Khodary 2004). In addition, it is becoming clear that SA interacts both negatively and positively with other major signaling pathways, including those regulated by jasmonic acid and ethylene (Raskin 1992). Furthermore, it is demonstrated that SA is able to trigger nitric oxide synthesis in Arabidopsis seedlings. Studying the kinetics of accumulation of nitric oxide, a clear response was observed that was dependent on the concentration of SA (Zottini et al. 2007).

Nitric oxide (NO) is another signaling molecule, which plays an important role in many physiological processes in plants, such as growth, development, senescence and adaptive responses to multiple stresses (Hu et al. 2007; Farooq et al. 2009; Kazemi et al. 2010). Recently, it has been shown that treatment with NO or its donor can mediate chlorophyll increase, Fe availability and antioxidant enzymes (Zhang et al. 2012). Several models suggest that redox signalling through NO and ROS is enhanced by SA in a self-amplifying process. However, the relationship between NO, SA, and ROS in the activation of defence genes and/or induction of host cell death is not clearly defined (Zottini et al. 2007).

Iron deficiency has been a widespread problem in peanut (*Arachis hypogaea* L*.*) grown on calcareous soils of northern China and has resulted in significant yield losses (Gao and Shi 2007). Previous researches demonstrated that the interaction of SA with SNP could counteract the deleterious effects induced by NaCl (Simaei et al. 2011). Based on the above studies, we suppose that SA and NO participates in Fe deficiency tolerance was important in peanut seedlings and the interaction of SA with NO could ameliorate Fe deficiency induced chlorosis. Therefore, the purpose of the present study was to examine whether foliar application of SA and SNP could (i) improve Fe uptake, transportation and activation; (ii) modulate the ions balance; (iii) protect Fe deficiency induced oxidative stress.

## Methods

### Plant materials and experimental design

The selected seeds of peanut (*Arachis hypogaea* L.) were surface sterilized with 5% H_2_O_2_ for 30 min, washed several times with sterile deionized water, and germinated on wet sterile sand at 25°C in the darkness. After germination, uniform seedlings transferred into aerated one-half strength Hoagland solution for 3 days. Then, the solution was replaced with standard Hoagland solution (Hoagland and Arnon 1950). When the third leaf expanded fully, the seedlings were transferred into hydroponic systems (Hoagland nutrient solution which substituting 0.1 mM FeSO_4_ for 0.1 mM EDTA-Fe) and removed cotyledons. Six treatments were established as follows: (1) hydroponic systems (CK); (2) Hoagland nutrient solution containing 0.1 mM EDTA-Fe (EDTA-Fe); (3) hydroponic systems and foliar application of 1.0 mM SA (SA); (4) hydroponic systems and foliar application of 2.5 mM SNP (SNP); (5) hydroponic systems and foliar application of 1.0 mM SA and 2.5 mM SNP (SA+SNP); (6) hydroponic systems and foliar application of 0.5 mM SA and 1.25 mM SNP [1/2(SA+SNP)]. The treatments are arranged in a randomized block design with three replicates. Treatments of spraying SA and/or SNP solution on leaves were performed in the evening every day with 10 mL every time. Each pot had 2.5 L of aerated nutrient solution and contained four plants. Nutrient solutions were replaced every three days and initial pH value was adjusted to 6.3 with 1.0 mM NaOH. Plants were grown in a growth chamber at 25 ± 1°C/20 ± 1°C (day/night) temperatures with a 16 h photoperiod at a light intensity of 300 ± 10 μEm^-2^ s^-1^ provided by reflector sunlight dysprosium lamp. Relative humidity ranged from 65 to 70%.

### Determination of Chlorophyll content, photosynthetic parameters and fluorescent parameters

The chlorophyll content was determined according to the method of Knudson et al. (1977). 0.5 g fresh peanut leaves were extracted in 2 mL 95% (v/v) ethanol for 24 h in the dark, and the extracted solution was analyzed. The amounts of chlorophyll *a*, *b* and carotenoid were determined spectrophotometrically (SHIMADZU UV-2450, Kyoto, Japan), by reading the absorbance at 665, 649 and 470 nm. C_a_ = 13.95*A665-6.88*A649; C_b_ = 24.96*A649-7.32*A665; C_carotenoid_ = (1000*A470-2.05*C_a_-114.8*C_b_)/245. The chlorophyll content results are expressed as unit’s mg per gram-fresh weight (mg·g^-1^ FW).

The young leaves were selected to measure photosynthetic and fluorescent parameters between 9:00–10:00 AM by using the photosynthesis system (CIRAS-2, UK) and the pulse amplitude modulated system (model FMS2. Hansatech Instruments. UK).

### Determination of the nutrient solution pH

The nutrient solution pH was determined with a MV-pH meter (DMP-2), and each treatment was replicated for three times. From the nineteen day after establishing Fe deficiency condition, in other words, from the last time change nutrient solutions, the nutrient solution pH was measured from 8 a.m. to 6 p.m. for every two hours in the first day (nineteen-day-old peanut seedlings), and measured at 2 p.m. in the second (twenty-day-old peanut seedlings) and third day (twenty one-day old seedlings). During the measuring period, the nutrient solution was not replaced, and only distilled water was added to replenish that lost by evaporation.

### Isolation of plasma membrane and the measurement of H^+^-ATPase and Ca^2+^-ATPase activities in PMs

A membrane fraction enriched in plasma membrane vesicles was prepared as described by Briskin et al. (1987) with minor changes. Excised roots were homogenized (1/2, w/v) with a mortar and pestle in a cold grinding medium containing: 25 mM HEPES-Tris (pH 7.2), 250 mM mannitol, 5 mM EDTA, 5 mM EGTA, 1 mM DTT and 1.5% (w/v) PVP. The whole isolation procedures were carried out at 4°C. The homogenate was filtered through four layers of cheesecloth and centrifuged at 560 × g for 12 min, then the supernatant was centrifuged at 10,000 × g for 15 min, and the supernatant was centrifuged at 60,000 × g for 30 min to yield a crude membrane fraction. The resulting pellet was resuspended with 1 mL in a gradient buffer containing: 20 mM HEPES-Tris (pH 7.5), 5 mM EDTA, 0.5 mM EGTA. The supernatant was layered on top of a step gradient consisting of 1 mL 45%, 33%, and 15% (w/w) sucrose, respectively, and then centrifuged for 2 h at 70,000 × g.

ATP hydrolysis assays were performed as described by Briskin et al. (1987) in 0.5 mL the reaction medium containing: 36 mM Tris-Mes (pH 6.5), 30 mM ATP-Na_2_, 3 mM MgSO_4_, 1 mM NaN_3_, 50 mM KNO_3_, 1 mM Na_2_MoO_4_, 0.02% (w/v) Triton A-100, in the presence or absence of 2.5 mM Na_3_VO_4_. The reaction was started by adding 50 μL PM vesicles. After 30 min incubation at 37°C, the reaction was quenched by the addition of 50 μL 55% (w/v) TCA. The H^+^-ATPase activity was determined by measuring the release of Pi (Ohinishi et al. 1975).

Additionally, ATP hydrolysis assays were performed as described by Briskin et al. (1987) in 0.5 mL the reaction medium containing: 50 mM Tris-Me (pH 7.6), 250 mM sucrose, 1.0 mM DTT, 2.0 mM ATP-Na_2_, 0.1 mM (NH_4_)_2_MoO_4_, 0.02% TritonX-100 (v/v), 300 Mm NaNO_3_, 1.0 mM NaN_3_, in the presence or absence of 2.0 mM Ca(NO_3_)_2_. The reaction was started by adding 50 μL PM vesicles. After 30 min incubation at 37°C, the reaction was quenched by the addition of 50 μL 55% (w/v) TCA. The Ca^2+^-ATPase activity was determined by measuring the release of Pi (Ohinishi et al. 1975).

Soluble protein content was measured by the Coomassie Brilliant Blue G-250 method (Bradford, 1976). Soluble protein content was expressed as mg g^-1^ FW.

### Assay of Fe(III)-Chelate Reductase (FCR)

Plants roots were immersed in saturated CaSO_4_ solution for 5 min, washed with deionized water, and then transferred to the nutrient solution mentioned above, which contained 0.1 mM Fe-EDTA and 0.4 mM 2, 2-bipyridyl, pH 5.0. The environmental conditions during the measurement were the same as for plant growth. After 2 h, Fe-reduction capacity by the roots was determined by measuring the concentration of Fe^2+^-dipyridyl complex formed at A_523_ in a spectrophotometer (Shimadzm UV-2210) (Gao and Shi 2007).

### Determination of active Fe content

Fresh young leaves were cut into pieces and 2.00 g was weighed and extracted with 20 mL 1 N HCl (in 1:10 tissue: extractant), shaken for 5 h and filtered, and the subsequent Fe concentration in the filtrate was measured with atomic absorption spectrophotometer (PE-2100B, Perkin Elmer Co. Ltd., MA, USA) (Gao and Shi 2007).

### Determination of the distribution of Fe in subcellular structure of peanut plants

After 21 days of treatment, roots of all the peanut plants were immersed in 20 mM disodium ethylenediamine tetraacetic acid (Na_2_-EDTA) for 15 min and were then rinsed three times with deionized water. Each 25 g sample was homogenized in 50 mL of chilled extraction buffer containing 50 mM Hepes (pH 7.5), 500 mM sucrose, 1 mM DTT, 5 mM ascorbate and 1% polyvinylpolypyrrolidone (PVPP). The homogenate was centrifuged at 500 × g for 5 min to isolate the cell wall fraction. The supernatant from this centrifugation step was then centrifuged at 20,000 × g for 45 min to sediment cell organelles, and the resultant supernatant solution was referred to as the soluble fraction. All steps were performed at 4°C. The fractions of the samples were digested in a mixture of HNO_3_ and HClO_4_ (4:1, v/v) at 120°C for at least 3 h. Fe was then quantified using an atomic absorption spectrometer (AA-6800, Shimadzu, Tokyo, Japan).

### Antioxidant enzymes and MDA extraction and assay

For determination antioxidant enzymes, 0.5 g powder of freeze-dried peanut leaves were homogenized with 50 mM Na_2_HPO_4_-NaH_2_PO_4_ buffer (pH 7.8) containing 0.2 mM EDTA and 2% insoluble polyvinylpyrrolidone in a chilled pestle and mortar. The slurry was centrifuged at 12,000 × g for 20 minutes and the supernatant was used for enzyme activities assay. The experiment was carried out at 4°C.

Total SOD activity was assayed by the photochemical method (Rao and Sresty 2000). The 3 mL reaction mixture contained 50 mM phosphate buffer (pH 7.8), 13 mM methionine, 75 μM nitroblue tetrazolium, 2 mM riboflavin, 10 mM EDTA and 0.1 mL enzyme extract. One unit of the enzyme activity was defined as the amount of enzyme required to result in a 50% inhibition of the rate of nitro blue tetrazolium (NBT) reduction measured at 560 nm. SOD activity was expressed as U g^-1^ FW min^-1^. Guaiacol peroxidase (POD) activity was estimated after Nickel and Cunningham (1969) method. Activity was measured by the increase in absorbance at 470 nm due to guaiacol oxidation. The activity was expressed as U g^-1^ FW min^-1^. CAT activity was determined by following the consumption of H_2_O_2_ at 240 nm according to the method of Cakmak and Marschner (1992). CAT activity was expressed as nmol H_2_O_2_ mg^-1^ FW min^-1^. The level of lipid peroxidation in fresh leaf was measured in terms of malondialdehyde (MDA) content by the thiobarbituric acid reaction method (Heath and Packer 1968). MDA content was expressed as nmol g^-1^ FW.

### Determination of the O_2_^•-^ generation rate

For the measurement of O_2_^•-^ germination rate, 0.3 g fresh leaves were ground in liquid N_2_ and extracted in 3 mL of ice cold 50 mM sodium phosphate buffer (PBS) (pH 7.0). One milliliter supernatant of fresh leaves extraction was added to 0.9 mL 65 mM phosphate buffer solution (pH 7.8) and 0.1 mL 10 mM hydroxylammoniumchloride. The reaction was incubated at 25°C for 35 min. 0.5 mL solution from the above reaction mixture was then added in 0.5 mL 17 mM sulfanic acid and 0.5 mL 7.8 mM ɑ-naphthylamine solution. After 20 min of reaction, 2 mL of ether was added into the above solution, and then mixed well. The solution was centrifuged at 1500 × g at 4°C for 5 min. The absorbance of the pink supernatant was measured at 530 nm with the spectrophotometer. Absorbance values were calibrated to a standard curve generated with known concentrations of HNO_2_ (Shi and Zhu 2008).

### Determination of plant growth index and mineral element concentrations

After 21 days treatment, the peanut plants were harvested. Roots were soaked in 20 mM Na_2_-EDTA for 15 min to remove metal ions adhering to root surfaces, and rinsed with deionized water several times. The peanut plants were divided into roots and shoots and measured plant height, root length and fresh weight. The root volume was determined by the water. The samples were dried at 70°C to constant weight and weighted. 50 mg of dried plant tissues were ground up and digested in 1 mL of concentrated nitric acid for 2–3 d at room temperature. Samples were then boiled for 1–2 h until completely digested. After adding 4 mL of millipore-filtered deionized water and briefly centrifuging, the mineral element concentrations of each sample were determined by atomic absorption spectrophotometer (SHIMADZU AA-6300) (Zhang et al. 2011).

### Statistical analysis

The tukey’s test was used to test the effect of different treatment, and the least significant difference (LSD) was calculated to compare the difference between means in each treatment. Statistical analyses were performed by analysis of variance (ANOVA) using the SAS software and correlative analysis used the SPSS software (SPSS 11.5).

## Results

### Plant growth

Table 1 shows that plant growth characteristics of peanut were greatly influenced by Fe deficiency and foliar application of SA and/or SNP. Fe deficiency dramatically decreased the shoot height, fresh weight and root/shoot ratio. However, addition of SA, SNP, especially SA+SNP alleviated the Fe deficiency effects. When peanut plants were treated with SA or SNP under Fe deficiency, the shoot height, root length and root volume in SA treatment increased by 18.2%, 29.3% and 19.4% compared with control, and the fresh weight and root/shoot ratio in SNP treatment increased by 28.4% and 33.3% compared with control. In addition, the alleviation effect of SA+SNP was detected best compared with SA, SNP or 1/2 (SA+SNP) treatment.

**Table 1:** **Effects of exogenous SA and NO on plant growth of peanut at 21 days treatment**

Treatments	Shoot height (cm/plant)	Root length (cm/plant)	Root volume (mL/plant)	Fresh weight (g/plant)	Root/Shoot ratio
CK	28.60 ± 0.66c	8.53 ± 0.06d	0.98 ± 0.08d	6.17 ± 0.08d	0.18 ± 0.01d
EDTA-Fe	33.90 ± 0.36b	9.63 ± 0.78d	1.31 ± 0.01b	7.05 ± 0.70c	0.21 ± 0.01c
SA	33.80 ± 0.79b	11.03 ± 1.23c	1.17 ± 0.06c	7.50 ± 0.66bc	0.22 ± 0.01bc
SNP	34.33 ± 0.46b	11.67 ± 0.38bc	1.18 ± 0.07c	7.90 ± 0.29ab	0.24 ± 0.01b
SA+SNP	36.77 ± 0.57a	13.23 ± 0.32a	1.49 ± 0.05a	8.29 ± 0.23a	0.26 ± 0.01a
1/2(SA+SNP)	34.50 ± 0.46b	12.73 ± 0.59ab	1.37 ± 0.08b	8.16 ± 0.13ab	0.25 ± 0.01a

CK: hydroponic systems; EDTA-Fe: Hoagland nutrient solution containing 0.1 mM EDTA-Fe; SA: hydroponic systems and foliar application of 1.0 mM SA; SNP: hydroponic systems and foliar application of 2.5 mM SNP; SA+SNP: hydroponic systems and foliar application of 1.0 mM SA and 2.5 mM SNP; 1/2(SA+SNP): hydroponic systems and foliar application of 0.5 mM SA and 1.25 mM SNP. Different lower case letters in the same line indicate a significant difference (P < 0.05) between different treatments. Data are means ± SD of three replicate samples per treatment.

### Chlorophyll content

As shown in Table 2, there was a significant decrease on chlorophyll content of peanut plants under Fe deficiency. However, foliar application of SA, SNP, 1/2(SA+SNP), especially SA+SNP, the inhibitory effects of Fe deficiency on chl *a* and chl *b* were significantly ameliorated. Additionally, foliar application of SA+SNP dramatically increased the chl *a*+*b*, car and chl *a/b* ratio by 19.1%, 26.3% and 3.6% compared with control, respectively.

**Table 2:** **Effects of exogenous SA and NO on** ***chl*** **a,** ***chl*** **b, car,** ***chl*** **a+b and** ***chl*** **a/b of peanut**

Treatments	Chl ***a***(mg g^-1^FW)	Chl ***b***(mg g^-1^FW)	Carotenoids ***x.c***(mg g^-1^FW)	Chl ***a+b***(mg g^-1^FW)	Chl ***a/b***
CK	1.15 ± 0.010f	0.46 ± 0.003d	0.19 ± 0.010b	1.62 ± 0.010f	2.49 ± 0.022b
EDTA-Fe	1.22 ± 0.003e	0.48 ± 0.002c	0.20 ± 0.009ab	1.70 ± 0.005e	2.51 ± 0.008ab
SA	1.26 ± 0.001d	0.50 ± 0.004bc	0.20 ± 0.011ab	1.76 ± 0.005d	2.53 ± 0.018ab
SNP	1.29 ± 0.011c	0.51 ± 0.020b	0.21 ± 0.028ab	1.79 ± 0.025c	2.55 ± 0.098ab
SA+SNP	1.39 ± 0.006a	0.54 ± 0.004a	0.24 ± 0.011a	1.93 ± 0.004a	2.58 ± 0.031a
1/2(SA+SNP)	1.35 ± 0.005b	0.53 ± 0.003a	0.22 ± 0.032a	1.88 ± 0.002b	2.56 ± 0.023ab

CK: hydroponic systems; EDTA-Fe: Hoagland nutrient solution containing 0.1 mM EDTA-Fe; SA: hydroponic systems and foliar application of 1.0 mM SA; SNP: hydroponic systems and foliar application of 2.5 mM SNP; SA+SNP: hydroponic systems and foliar application of 1.0 mM SA and 2.5 mM SNP; 1/2(SA+SNP): hydroponic systems and foliar application of 0.5 mM SA and 1.25 mM SNP. Different lower case letters in the same line indicate a significant difference (P < 0.05) between different treatments. Data are means ± SD of three replicate samples per treatment.

### Photosynthetic and fluorescent parameters

Peanut plants subjected to Fe deficiency exhibited a dramatic decrease in net photosynthetic (*Pn*) and transpiration rates (*Tr*) by 32.9% and 23.5% compared with EDTA-Fe treatment, whereas Fo, Fv/Fm and ΦPSII also significantly decreased in peanut leaves (Table 3). However, when peanut plants treated with SA, SNP, 1/2(SA+SNP), particularly SA+SNP, the inhibition of photosynthetic system induced by Fe deficiency remarkably alleviated. In addition, the Fo, Fv/Fm and ΦPSII of SA+SNP treatment, interestingly increased by 26.55%, 8.6% and 26.9% compared with control, and the Fv/Fm and ΦPSII were similar to EDTA-Fe treated ones.

**Table 3:** **Effects of exogenous SA and NO on the net photosynthetic (Pn), transpiration rate (Tr), Fo, Fv/Fm and ΦPSII of leaves**

Treatments	Pn [μmol m^-2^·s^-1^]	Tr [mmol m^-2·^s-^1^]	Fo	Fv/Fm	ΦPSII
CK	10.78 ± 1.14e	0.88 ± 0.11e	82.44 ± 1.71c	0.805 ± 0.04b	0.439 ± 0.02c
EDTA-Fe	16.07 ± 0.98d	1.15 ± 0.03d	95.78 ± 0.69b	0.873 ± 0.04a	0.535 ± 0.02a
SA	18.65 ± 0.86c	1.64 ± 0.07c	97.78 ± 1.07b	0.843 ± 0.04ab	0.483 ± 0.01b
SNP	18.72 ± 0.85c	1.79 ± 0.22c	97.67 ± 0.58b	0.858 ± 0.01a	0.468 ± 0.02b
SA+SNP	24.89 ± 1.24a	4.26 ± 0.15a	104.33 ± 3.79a	0.874 ± 0.01a	0.557 ± 0.02a
1/2(SA+SNP)	24.82 ± 0.65b	3.99 ± 0.18b	100.33 ± 4.51ab	0.865 ± 0.01a	0.466 ± 0.01bc

CK: hydroponic systems; EDTA-Fe: Hoagland nutrient solution containing 0.1 mM EDTA-Fe; SA: hydroponic systems and foliar application of 1.0 mM SA; SNP: hydroponic systems and foliar application of 2.5 mM SNP; SA+SNP: hydroponic systems and foliar application of 1.0 mM SA and 2.5 mM SNP; 1/2(SA+SNP): hydroponic systems and foliar application of 0.5 mM SA and 1.25 mM SNP. Different lower case letters in the same line indicate a significant difference (P < 0.05) between different treatments. Data are means ± SD of three replicate samples per treatment.

### The pH of nutrient solution and the activities of H^+^-ATPase and Ca^2+^-ATPase

**Figure 1:**
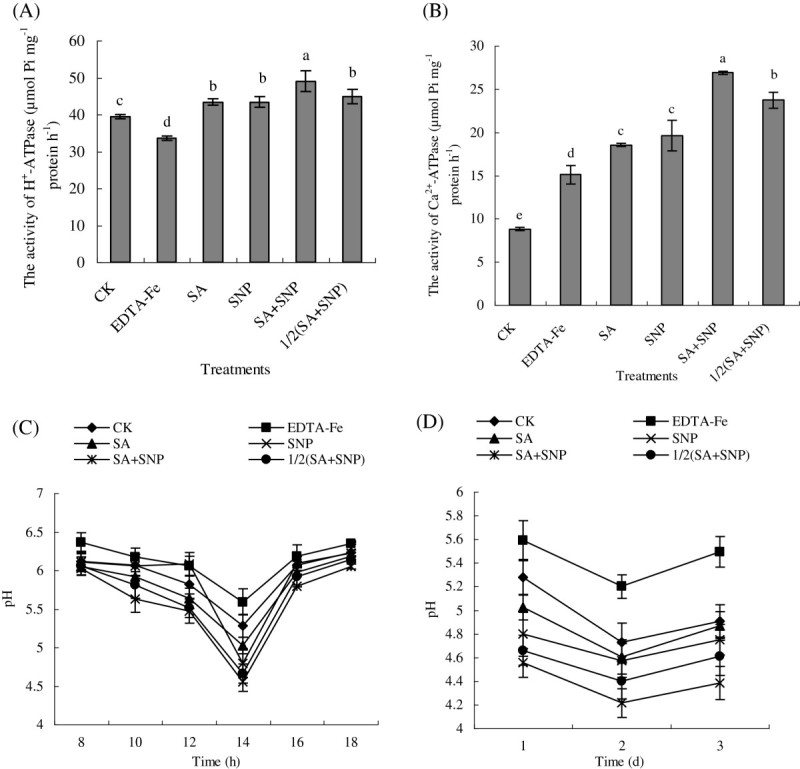
**Effects of exogenous SA and NO on the activities of plasma membrane H**^**+**^**-ATPase (A) and Ca**^**2+**^**-ATPase (B) in roots of peanut seedlings and changes of pH in culture medium (C and D).** Different lower case letters in the same line indicate a significant difference (P < 0.05) between different treatments. The values are means ± SD of three replicate samples per treatment. CK: hydroponic systems; EDTA-Fe: Hoagland nutrient solution containing 0.1 mM EDTA-Fe; SA: hydroponic systems and foliar application of 1.0 mM SA; SNP: hydroponic systems and foliar application of 2.5 mM SNP; SA+SNP: hydroponic systems and foliar application of 1.0 mM SA and 2.5 mM SNP; 1/2(SA+SNP): hydroponic systems and foliar application of 0.5 mM SA and 1.25 mM SNP.

The H^+^-ATPase transports protons out of the cell across the plasma membrane, thus establishing the proton electrochemical gradient that contributes to the maintenance of the intracellular and extracellular pH and plays a major role in the activation of ion and nutrient transport (Frédéric et al. 2007). Therefore, we determined the activities of H^+^-ATPase and Ca^2+^-ATPase in plasma membrane and pH of nutrient solution. As shown in Figure 1, under Fe deficiency, the activity of H^+^-ATPase was considerably stimulated. Compared with the control, SA+SNP and 1/2(SA+SNP) treatments further increased the activity of H^+^-ATPase by 24.39% and 13.88%, respectively. Fe deficiency inhibited the activity of Ca^2+^-ATPase. However, the Ca^2+^-ATPase activity was significantly increased by foliar application of SA, SNP, or 1/2(SA+SNP), especially SA+SNP. In addition, as shown in Figure 1C and D, on the whole, the nutrient solution pH of all treatments exhibited a decline from 8 a.m. to 14 p.m. in the first day, although it appeared to fluctuate in the decline. Under Fe-deficiency stress, pH value in nutrient solution was significantly lower than EDTA-Fe treatment. SA+SNP treatment, in particular, showed the lowest pH at 14 p.m. in the first day. Furthermore, on the whole, the nutrient solution pH of all treatments exhibited a decline from the first day to the second day, and an increase from the second day to the third day (Figure 1B).

### Total Fe, active Fe and FCR activity

As shown in Table 4, Fe deficiency inhibited Fe absorption and transportation of peanut seedlings, producing a 38.4% (roots), a 31.3% (stems) and a 19.5% (leaves) decrease in the Fe concentrations compared with EDTA-Fe treatment. Moreover, it markedly decreased the active Fe content. However, it strikingly increased FCR activity compared with EDTA-Fe treatment. Foliar application of SA, SNP or 1/2(SA+SNP), particularly SA+SNP showed a positive influence on Fe concentrations, active Fe content, and FCR activity. SA+SNP significantly increased the Fe concentrations in roots, stems and leaves by 37.68%, 55.40% and 111.75%, and dramatically increased the active Fe content and FCR activity by 91.5% and 28.2%, respectively, compared with control.

**Table 4:** **Effects of exogenous SA and NO on the Fe concentration, active Fe content and Fe (III)- chelate reductase activity**

Treatments	Fe concentration (mg kg^-1^DW)			Active Fe content (mg kg^-1^FW)	Fe(III)- chelate reductase acticity (μmol g^-1^2 h^-1^FW)
	Roots	Stems	Leaves		
CK	852.07 ± 2.92e	118.33 ± 9.09d	125.11 ± 9.20e	33.45 ± 1.10f	3.12 ± 0.09d
EDTA-Fe	1382.71 ± 8.49a	172.34 ± 8.52b	155.36 ± 2.48d	50.02 ± 0.81e	2.82 ± 0.09e
SA	865.35 ± 18.99e	160.84 ± 4.47c	159.24 ± 9.64d	51.56 ± 0.25d	3.35 ± 0.02c
SNP	916.50 ± 2.89d	168.33 ± 4.62bc	183.34 ± 5.20c	53.42 ± 0.56c	3.37 ± 0.07c
SA+SNP	1173.14 ± 12.91b	183.89 ± 4.61a	264.92 ± 4.63a	64.06 ± 0.49a	4.00 ± 0.12a
1/2(SA+SNP)	1022.90 ± 8.78c	173.96 ± 5.12ab	227.15 ± 9.77b	61.30 ± 0.70b	3.74 ± 0.06b

CK: hydroponic systems; EDTA-Fe: Hoagland nutrient solution containing 0.1 mM EDTA-Fe; SA: hydroponic systems and foliar application of 1.0 mM SA; SNP: hydroponic systems and foliar application of 2.5 mM SNP; SA+SNP: hydroponic systems and foliar application of 1.0 mM SA and 2.5 mM SNP; 1/2(SA+SNP): hydroponic systems and foliar application of 0.5 mM SA and 1.25 mM SNP. Different lower case letters in the same line indicate a significant difference (P < 0.05) between different treatments. Data are means ± SD of three replicate samples per treatment.

### The distribution of Fe in subcellular structure of peanut plants

**Figure 2:**
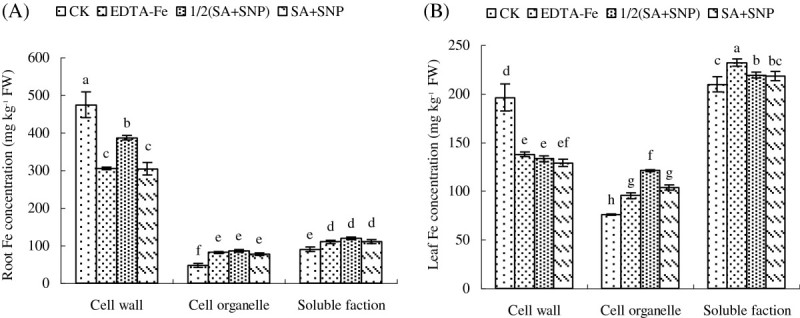
**Effects of different treatments on tissue distribution of Fe in peanut roots (A) or leaves (B).** Different lower case letters in the same line indicate a significant difference (P < 0.05) between different treatments. The values are means ± SD of three replicate samples per treatment. CK: hydroponic systems; EDTA-Fe: Hoagland nutrient solution containing 0.1 mM EDTA-Fe; SA: hydroponic systems and foliar application of 1.0 mM SA; SNP: hydroponic systems and foliar application of 2.5 mM SNP; SA+SNP: hydroponic systems and foliar application of 1.0 mM SA and 2.5 mM SNP; 1/2(SA+SNP): hydroponic systems and foliar application of 0.5 mM SA and 1.25 mM SNP.

In order to investigate the mechanisms of exogenous SA and SNP in alleviating chlorosis induced by Fe deficiency, Fe distributions in cell wall, soluble fraction and cell organelles were examined carefully. In the roots, majority of Fe accumulated in the cell wall. However, Fe accumulated in the soluble fraction was less than in the cell wall, and only a minority of Fe accumulation was in cell organelles (Figure 2A). In contrast, in the leaves, the amount of Fe accumulated in the soluble fraction was more than the amount of Fe accumulated in the cell wall, and the cell organelles accumulated the least Fe (Figure 2B). The results were quite different in the plants treated with Fe deficiency and foliar application of SA+SNP. Under Fe deficiency, Fe concentrations were significantly increased in the cell wall, but dramatically decreased in the cell organelle and soluble fraction in the roots and leaves of peanut. Foliar application of SA+SNP, Fe concentrations in the roots and leaves were interestingly increased in the cell organelle and soluble fraction, but decreased in the cell wall. In addition, the effect of SA+SNP was better than 1/2(SA+SNP).

### Antioxidant enzymes, MDA and O_2_^•-^ generation rate

Results in Table 5 demonstrated that Fe deficiency led to the MDA content and O_2_^•-^ generation rate increased dramatically in the cells of peanut leaves. Foliar application of SA and/or SNP, the oxidative damage induced by Fe deficiency was significantly alleviated. As we all know, SOD, POD and CAT are all important antioxidant enzymes. Foliar application of SA, SNP, or 1/2(SA+SNP), especially SA+SNP, significantly increased the activities of these enzymes. Foliar application of SA+SNP significantly increased the activities of SOD, POD and CAT by 9.32%, 113.3% and 22.41%, respectively, compared with control.

**Table 5:** **Effects of exogenous SA and NO on SOD, POD and CAT activities and MDA content and superoxide produce rate of peanut leaves**

Treatments	SOD activity (Unit mg^-1^protein)	POD activity (Unit mg^-1^protein)	CAT activity (Unit g^-1^protein)	MDA content (nmol g^-1^FW)	O_2_^·-^generation rate (μmol g^-1^ h^-1^FW)
CK	2.36 ± 0.08b	0.30 ± 0.04d	0.058 ± 0.003c	34.12 ± 3.98a	6.41 ± 0.52a
EDTA-Fe	2.34 ± 0.07b	0.41 ± 0.03c	0.067 ± 0.002b	19.21 ± 1.64b	4.73 ± 0.47bc
SA	2.52 ± 0.06a	0.47 ± 0.01c	0.069 ± 0.001ab	21.99 ± 3.71b	5.28 ± 0.06ab
SNP	2.52 ± 0.16a	0.50 ± 0.09bc	0.068 ± 0.001ab	19.93 ± 1.93b	4.55 ± 1.31bc
SA+SNP	2.58 ± 0.03a	0.64 ± 0.04a	0.071 ± 0.001a	17.31 ± 0.87b	3.50 ± 0.32c
1/2(SA+SNP)	2.52 ± 0.05a	0.57 ± 0.06ab	0.069 ± 0.002ab	18.77 ± 2.67b	4.40 ± 1.53bc

CK: hydroponic systems; EDTA-Fe: Hoagland nutrient solution containing 0.1 mM EDTA-Fe; SA: hydroponic systems and foliar application of 1.0 mM SA; SNP: hydroponic systems and foliar application of 2.5 mM SNP; SA+SNP: hydroponic systems and foliar application of 1.0 mM SA and 2.5 mM SNP; 1/2(SA+SNP): hydroponic systems and foliar application of 0.5 mM SA and 1.25 mM SNP. Different lower case letters in the same line indicate a significant difference (P < 0.05) between different treatments. Data are means ± SD of three replicate samples per treatment.

### Mineral element contents

**Figure 3:**
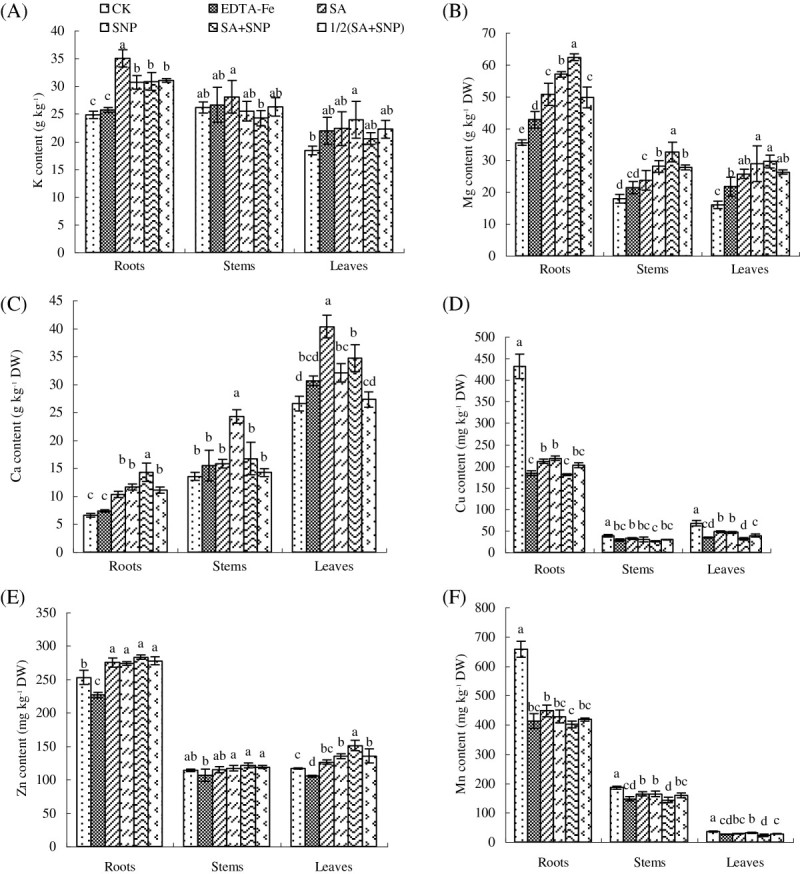
**Effects of exogenous SA and NO on the concentrations of K (A), Mg (B), Ca (C), Cu (D), Zn (E), and Mn (F) of peanut.** Different lower case letters in the same line indicate a significant difference (P < 0.05) between different treatments. The values are means ± SD of three replicate samples per treatment. CK: hydroponic systems; EDTA-Fe: Hoagland nutrient solution containing 0.1 mM EDTA-Fe; SA: hydroponic systems and foliar application of 1.0 mM SA; SNP: hydroponic systems and foliar application of 2.5 mM SNP; SA+SNP: hydroponic systems and foliar application of 1.0 mM SA and 2.5 mM SNP; 1/2(SA+SNP): hydroponic systems and foliar application of 0.5 mM SA and 1.25 mM SNP.

As shown in Figure 3, Fe deficiency dramatically disturbed the ionic homeostasis, leading to the concentrations of manganese (Mn), copper (Cu) and zinc (Zn) significantly increased, and the concentration of magnesium (Mg) interestingly decreased in roots. Foliar application of SA and/or SNP, the concentrations of Cu and Mn significantly decreased, however, the concentrations of Zn and Mg dramatically increased in the roots and leaves compared with control. SA+SNP increased the concentrations of potassium (K), Mg, calcium (Ca) and Zn by 11.4%, 85.9%, 30.6% and 19.0%, and decreased the concentrations of Cu and Mn by 53.1% and 34.9% compared with control in the leaves of peanut.

## Discussion

Symptoms of Fe deficiency in peanut have been previously described by Zuo et al. (2000). For example, Fe deficiency can cause decreased growth, alter photosynthetic rates and cause morphological changes in the leaves (Sun et al. 2007; Zhang et al. 2012). In this experiment, Fe deficiency-induced chlorophyll and carotenoids deficiency was closely correlated with visual observations of chlorosis of peanut leaves (Table 2), and similar results have been obtained in many other crop plants, such as pear (Morales et al. 2000), peach (Molassiotis et al. 2005), maize (Sun et al. 2007), and Chinese cabbage (Ding et al. 2008). The chlorosis can be ascribed to the role of Fe in the formation of δ-aminolevulinic acid and protochlorophyllide, the precursors of the chlorophyll biosynthesis. In addition, Fe deficiency led to an inhibitory effect on plant growth (Table 1). The inhibition of growth in peanut might be resulted from Fe deficiency-induced alteration of fundamental metabolic process, photosynthesis, active Fe content and uptake of nutrient elements.

SA and NO are participating in the regulation of multiple physiological processes and plant resistance to biotic and abiotic stress. Recently, it was established that NO elevates the content of SA known as a component of signaling pathways during biotic stresses (Klessig et al. 2000) and SA may in turn stimulate synthesis of NO in Arabidopsis thaliana, acting via the enzyme with NO synthesizing activity (Zottini et al. 2007). The effects of SA and NO on alleviating environment toxicity in plants, such as nickel and salt stress, has been reported by Kazemi et al. (2010) and Simaei et al. (2011). Therefore, in the experiment, we analyzed the possible role of foliar application of SA and/or SNP in modulation of the photosynthetic performance and the antioxidant defense system against Fe deficiency induced chlorosis in peanut seedlings. Results from this experiment clearly indicated that SA, SNP, particularly SA+SNP, effectively alleviated chlorosis induced by Fe deficiency, described as SA and SNP increased the uptake and translocation of Fe, promoted the activation of Fe in the leaves of peanut and protected Fe deficiency induced oxidative stress, resulting in increased chlorophyll content and improved seedling growth.

A notable reduction of chlorophyll parameters was detected in peanut seedlings exposed to Fe deficiency stress (Table 2; Zhang et al. 2012). The decrease in chlorophyll content might be attributed to Fe deficiency reduced active Fe content (Table 4), where active Fe participates in various physiological actions inside the plants, such as the electron transport, chlorophyll biosynthesis (Hakan and Vahap 2007; Zhang et al. 2011). Foliar application of SA, SNP, or 1/2(SA+SNP), especially SA+SNP to plants significantly enhanced the chlorophyll content in young leaves (Table 2; Kazemi et al. 2010). This phenomenon may be attributed to the fact that foliar application of SA and/or SNP decreased chlorophyll degradation caused by senescence and environmental stress. In addition, foliar application of SA+SNP, the active Fe content was dramatically increased in leaves (Table 4) and the Fe concentration in the cell organelle in leaves was also significantly increased (Figure 2). It indicated that SA+SNP can improve Fe enter into cell and promote the activation of Fe in the leaves of peanut. The production of NO has been considered as an early response to iron deprivation and is maintained while the plant is exposed to low-iron conditions (Graziano and Lamattina 2007). Chen et al. (2010) further demonstrated that NO acts as a signal downstream of the auxin under Fe deficiency that leads to the ultimate induction of FCR via FIT-mediated transcriptional regulation. Peanuts belong to Strategy I plants respond to Fe deficiency by inducing the ferric chelate reductase embedded in root epidermal cell plasma membrane and also via stimulation of the plasma membrane proton pump, thus increasing proton exudation (Vert et al. 2002; Jin et al. 2006). Therefore, the increased active Fe content may be because SA+SNP enhanced excretion of protons and increased activity of FCR to solubilize Fe^III^ oxides to Fe^II^ chelates. The export of protons from the plant cells is mediated by the plasma membrane H^+^-ATPase (Ligaba et al. 2004; Osses and Godoy 2006). One of the main functions of plasmalemma H^+^-ATPase is H^+^ extrusion from cell cytosol to cell wall area and loosening of cell wall microfibriles leading to cell growth by elongation (Hager 2003). These processes can be controlled by IAA and fusicoccine and possibly reveal the last phases of hormone controlled growth realization. In the experiment, the activities of H^+^-ATPase and FCR were significantly increased in roots by foliar application of SA and/or SNP, leading to the nutrient solution pH reduced and total Fe concentrations increased in roots. Moreover, the Fe concentration in the cell wall in roots was decreased and the Fe concentrations in the soluble fraction and cell organelles in roots were increased by foliar application of SA+SNP. In addition, foliar application of SA+SNP also dramatically increased the Fe concentrations in the roots, stems and leaves of peanut compared with control. Therefore, we can infer that SA+SNP improved the uptake and translocation of Fe. Since the monocropped peanut suffers from less Fe availability, peanut under monocropping changes the expression of Fe-responsive genes, such as AhIRT1, encoding an iron-uptake transporter (Ding et al. 2010), and AhFRO1, encoding ferric-chelate reductase (Ding et al. 2009). Peanut under Fe deficiency may be also changed AhIRT1 and AhFRO1 to improve Fe absorption and transportation in the experiment. However, when Fe availability is under a threshold level, those are not sufficient to support the Fe requirement for plant development, and the stress symptoms become evident. Exogenous application of SA+SNP increased the Fe concentrations in peanut roots, stems and leaves compared with Fe deficiency treatment. Whether this is because SA+SNP changed AhIRT1 and AhFRO1 improving Fe absorption and transportation or not is needed further research.

Fe deficiency significantly decreased the net photosynthesis (Table 3; Zhang et al. 2012). The factors mainly include stomatal limitation caused by stomatal closure and non-stomatal limitation. The former may be caused by several factors, including the decline of essential nutrient elements content, especially K, and the increased production of ROS in chloroplasts. The latter may be caused by Fe deficiency decreased active Fe content. Under our experimental conditions, the ionic homeostasis was greatly disturbed (Figure 3) and active Fe content was dramatically decreased (Table 4) by Fe deficiency. In addition, chlorophyll content and chlorophyll fluorescence are considered as indicators of damages to photosynthetic system induced by environmental stressors (Maxwell and Johnson 2000; Shi et al. 2009). As we all know, Fe is involved in chlorophyll biosynthesis. Therefore, Fe deficiency impairs chlorophyll biosynthesis and chloroplast development, where the photosynthesis in plants predominantly relies on the green leaf, and its productivity directly depends upon the chlorophyll bearing surface area, irradiance and its potential to utilize CO_2_. However, foliar application of SA and/or SNP stimulated the maintenance of ions disturbed by Fe deficiency. Bush (1993) has clearly established that Ca^2+^ acts as an intracellular messenger in coupling a wide range of extracellular signals to specific responses. In the present study, SA and/or SNP increased Ca^2+^-ATPase activity in roots and significantly increased Ca^2+^ concentration in leaves (Figure 3D). Earlier studies indicated that exogenous SA treatment stimulated root formation and increased mineral uptake by plants (Khan et al. 2003; Yildirim et al. 2008). In addition, SA has long been known as a signal molecule in the induction of defense mechanisms in plants (Horvath et al. 2007) and probably modulated the total amylase activity and a-amylase activity by hormonal regulation (He et al. 2010). This is why the role of SA on membrane leakage, lipid peroxidation, and mineral uptake under stress conditions is important (Gunes et al. 2007). This positive effect of SA and SNP could be attributed to enhanced CO_2_ assimilation, active Fe content, chlorophyll concentration, and photosynthetic rate, which protected photosynthesis system.

In plants, there are several reports which show metal-induced alterations in both the activities of antioxidative enzymes and the level of soluble antioxidants (Cakmak and Marschner 1992; Guo et al. 2007), accompanied by an enhancement of lipid peroxidation (Shi and Zhu 2008; Shi et al. 2009). Depending on its concentration, Fe deficiency can impair the electron transport and may lead to the production of ROS (Graziano and Lamattina 2005). The overproduction of ROS can lead to oxidative injury such as membrane lipid peroxidation, protein oxidation, enzyme inhibition and DNA and RNA damage (Mittler 2002). In our experiments, Fe deficiency-induced oxidative stress in peanut plants was evident to the increased MDA content and the O_2_^•-^ generation rate (Table 5). In normal physiological conditions, ROS keeps a dynamic balance between constantly produced and cleared. However, the balance was destroyed and the production of toxic oxygen derivatives was increased as a result of Fe deficiency stress. Fe deficiency increased SOD activity to convert the O_2_^•-^ radical to the H_2_O_2_. However, it decreased POD and CAT activities. POD and CAT are all heme-containing enzymes and their activities are likely to be affected by Fe deficiency. Therefore, in the study, SA and/or SNP increased active Fe content (Table 4) and also increased the activities of POD and CAT. SA and/or SNP treatment increased the activities of antioxidant enzymes and reduced the adverse effect of ROS on amylase activity under Fe deficiency stress. Our findings indicated that SA and SNP act as plant growth regulators that can alleviate oxidative damage induced by Fe deficiency. These results are in agreement with recent researches (Simaei et al. 2011). Foliar application of exogenous SA or SNP, it also could alleviate the Fe deficiency induced chlorosis. However, the effects of SA+SNP were better than SA or SNP alone, or 1/2(SA+SNP). This may be because SA+SNP made the concentration of SA and NO in the plant body achieve the best level, so as to considerably alleviated chlorosis induced by Fe deficiency.

## Conclusions

The present work demonstrates that SA and SNP can alleviate Fe deficiency-induced chlorosis separately or together. Additionally, the combination of SA and SNP in alleviating chlorosis is better than SA or SNP alone, but an appropriate concentration of SA and SNP was needed. The mechanism of SA and/or SNP alleviating Fe-deficiency induced chlorosis mainly contains: (1) promoted Fe uptake, translocation and activation, (2) modulated the balance of mineral elements, (3) protected Fe deficiency induced oxidative stress. Our results may serve as an integrated and optimized management strategy to alleviate Fe deficiency in peanuts production, but further study is needed.

## Authors’ original submitted files for images

Below are the links to the authors’ original submitted files for images. Authors’ original file for figure 1 Authors’ original file for figure 2 Authors’ original file for figure 3

## Abbreviations

Ca: Calcium
Car: Carotenoids
CAT: Catalase
Chl: Chlorophyll
Cu: Copper
FCR: Fe^(III)^-Chelate Reductase
Fe: Iron
Fo: The minimal fluorescence
Fv/Fm: Maximal quantum yield of PS II
MDA: Malondialdehyde
Mg: Magnesium
Mn: Manganese
O2• -: Superoxide anion
Pn: Net photosynthetic
POD: Peroxidases
ΦPSII: The effective quantum yield of PS II
SNP: Sodium nitroprusside
SA: Salicylic acid
SOD: Superoxide dismutase
Tr: Transpiration rates
Zn: Zinc.
