# Supplementary figures and images for: Effects of foliar application of salicylic acid and nitric oxide in alleviating iron deficiency induced chlorosis of Arachis hypogaea L
Source: Bot Stud. 2014 Jan 20;55:9. doi: 10.1186/1999-3110-55-9 (PMC5432746; doi:10.1186/1999-3110-55-9)

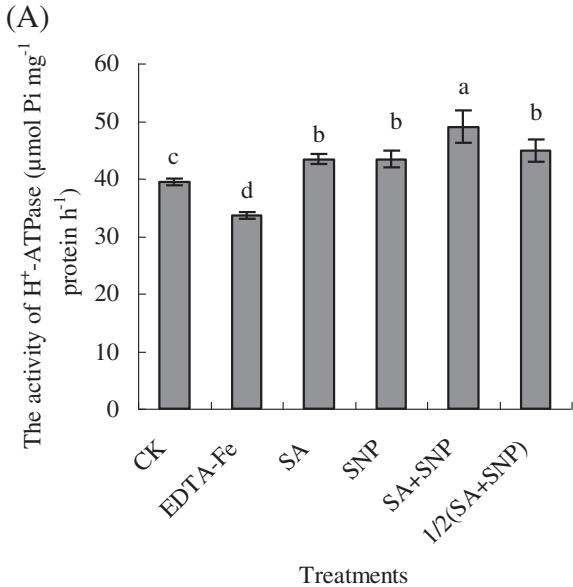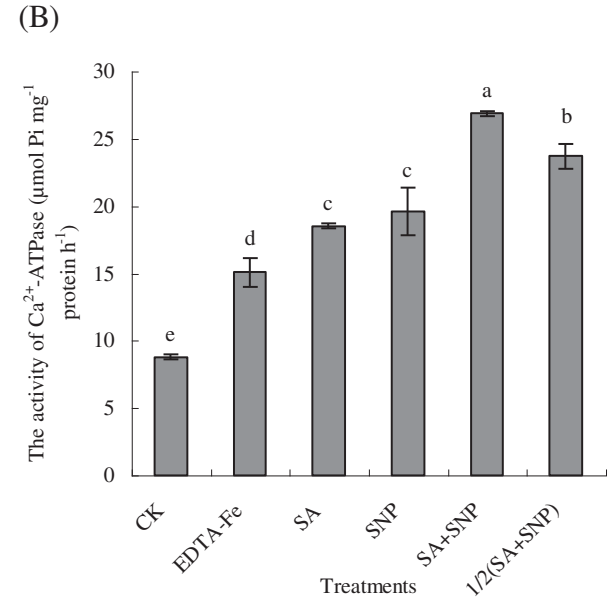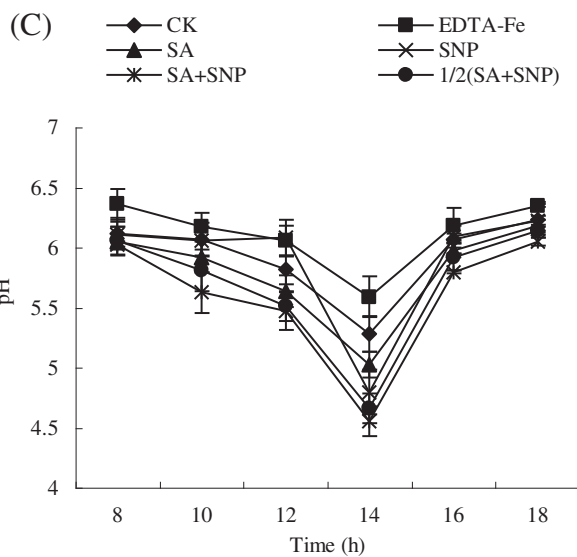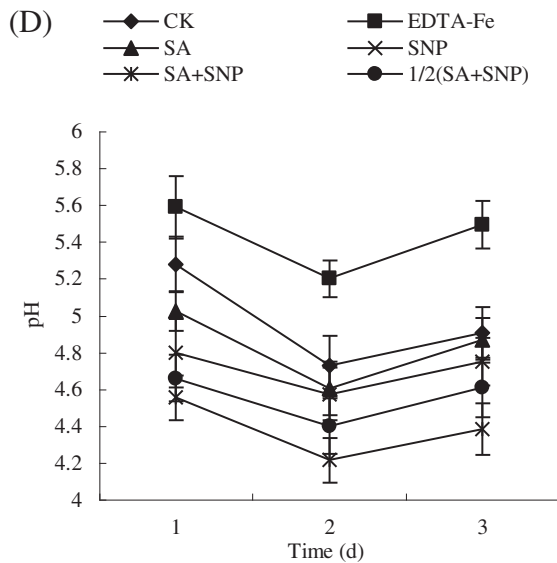

Supplement: Supplementary file 1 — Authors’ original file for figure 1 [file 40529_2013_60_MOESM1_ESM.pdf]

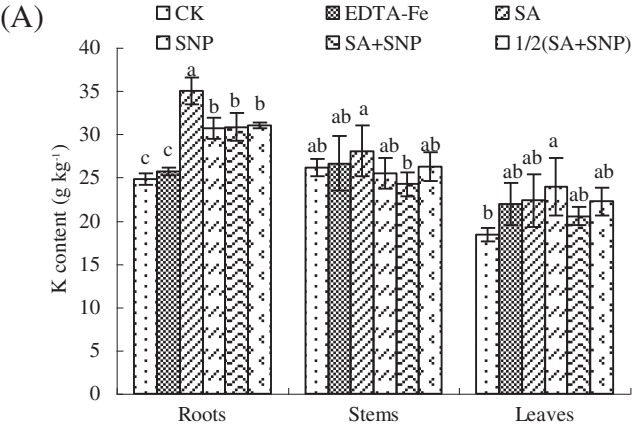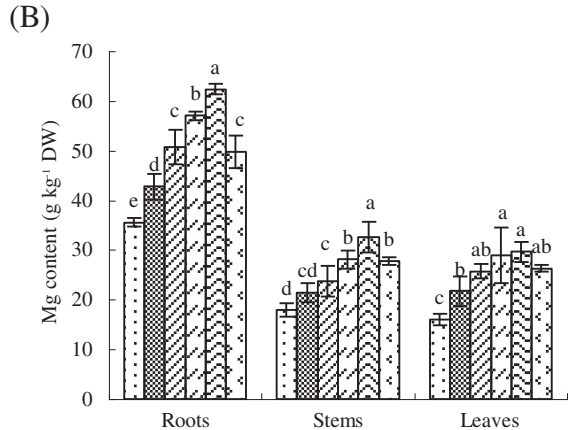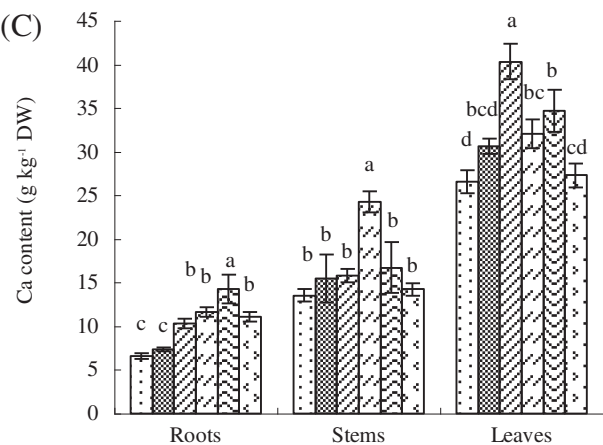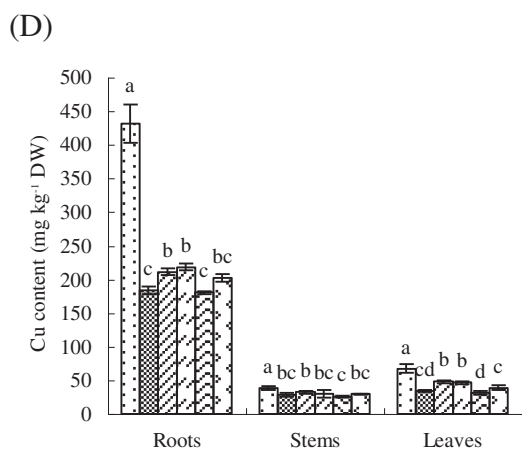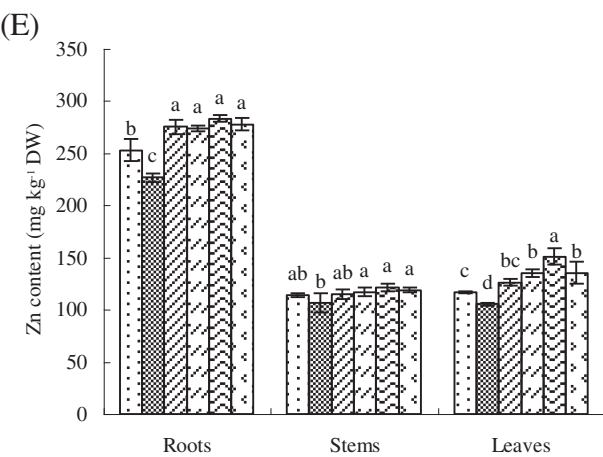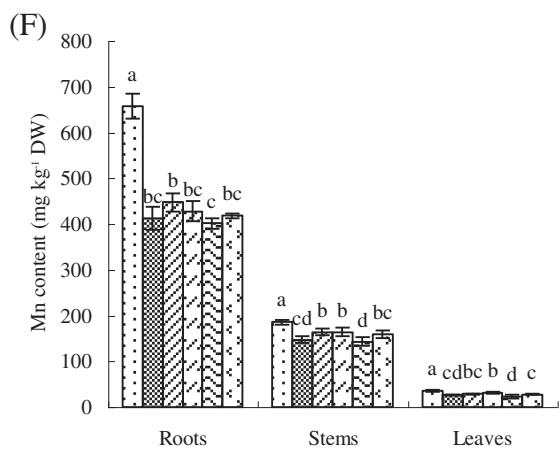

Supplement: Supplementary file 3 — Authors’ original file for figure 3 [file 40529_2013_60_MOESM3_ESM.pdf]
