# Supplementary material for: Effects of foliar application of salicylic acid and nitric oxide in alleviating iron deficiency induced chlorosis of Arachis hypogaea L
Source: Bot Stud. 2014 Jan 20;55:9. doi: 10.1186/1999-3110-55-9 (PMC5432746; doi:10.1186/1999-3110-55-9)
Supplement: Supplementary file 2 — Authors’ original file for figure 2 [file 40529_2013_60_MOESM2_ESM.pdf]

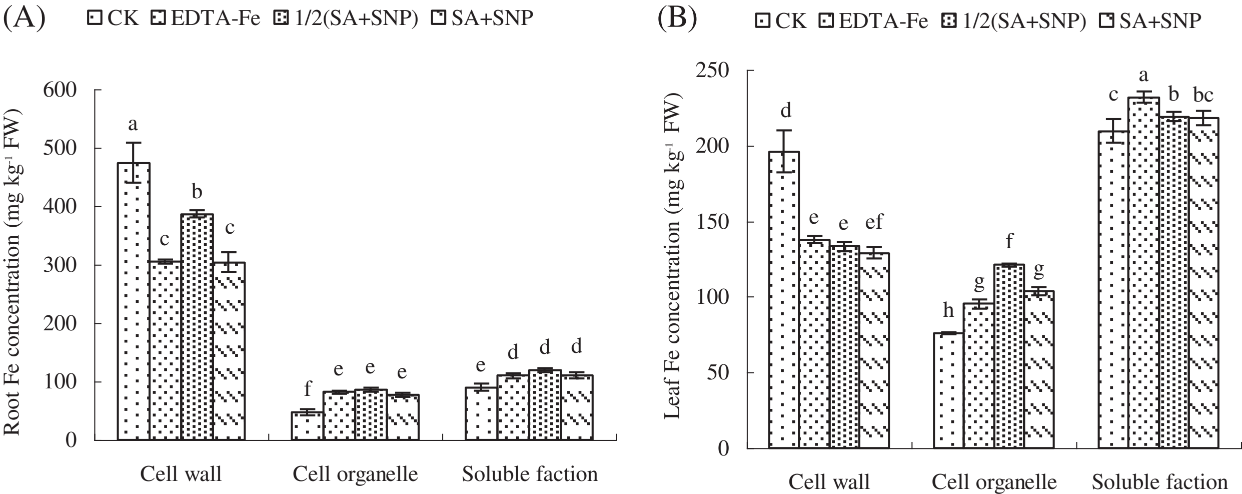
(A)
CK
EDTA-Fe
1/2(SA+SNP)
SA+SNP
Root Fe concentration (mg kg-1 FW)
0
100
200
300
400
500
600
a
c
b
c
f
e
e
e
e
d
d
d
Cell wall
Cell organelle
Soluble faction
(B)
CK
EDTA-Fe
1/2(SA+SNP)
SA+SNP
Leaf Fe concentration (mg kg-1 FW)
0
50
100
150
200
250
d
e
e
ef
h
g
f
g
c
a
b
bc
Cell wall
Cell organelle
Soluble faction
